# Delay to surgery beyond 12 hours is associated with increased hip fracture mortality

**DOI:** 10.1007/s00590-024-03997-5

**Published:** 2024-06-07

**Authors:** Madeline Warren, Chris Bretherton, Martyn Parker

**Affiliations:** 1 NWAFT, Peterborough, UK; 2 Queen Mary University London, London, UK; 3 Present Address: Bedfordshire Hospitals NHS Foundation Trust, Bedford, UK

**Keywords:** Hip fracture, Delay, Mortality

## Abstract

**Purpose:**

Time to surgery from admission is one of the few variables known to influence outcome after a hip fracture. We reviewed our hip fracture database to determine correlation between delays to surgery and mortality in our elderly hip fracture population.

**Methods:**

Data on all hip fracture patients admitted to a large district hospital were prospectively collected between January 1989 and August 2021. Time of the injury, time of admission and time of surgery were recorded. Patients over 60 years old with a hip fracture requiring operative management were included. Patients with pathological fractures, those managed conservatively, and patients delayed for medical reasons were excluded. Surgical timing categories were divided into; under 12 h, 12–24 h, 24–72 h and over 72 h.

**Results:**

Time from admission to surgery was recorded for 10,659 patients, of these time of fall was available for 10,346 patients. Mean age was 82.2 years (sd 8.39) for the cohort and 30 day mortality was 6.20%.

Odds of 30-day mortality was 1.43 (CI 1.057–1.988, *p* = 0.025) for delay to surgery from admission of over 12 h compared to under 12 h. Odds ratios for 30-day mortality were not significant at any other time threshold. The odds of 30-day mortality for delay to surgery from time of fall were 1.550 (CI 1.026–2.459, *p* = 0.048) at the 12 h threshold.

**Conclusion:**

This is the largest prospective study to date in elderly patients with hip fractures demonstrating a statistically significant increase in 30-day mortality with a delay to surgery over 12 h.

## Introduction

Over 70,000 patients are admitted to hospitals in England and Wales with hip fractures each year[1]. These injuries lead to high rates of morbidity and mortality amongst the elderly with rates of 30-day mortality of 6.6% prior to the 2020 covid-19 pandemic [1]. Many different factors have been proposed to impact on mortality, such as age and co-morbidities; however, the time delay to surgery remains one of the few variables that can be altered.

Various studies have reported differing time thresholds for significant improvements in mortality[2–15], with some finding no significant difference [16–21]. These studies use varying time cutoffs for analysing delay and mortality, and negative results were predominantly from smaller cohorts.

Whilst current UK guidelines recommend intervention within 36 h [22] evidence from our local cohort has previously shown benefit from operating earlier [23].

The aim of this study was to determine, using our final dataset, whether reducing the delay from admission to surgery continues to improve survivorship in our elderly hip fracture population and determine whether reducing delay from fall to surgery confers a similar survival benefit.

## Patients and methods

Data on all hip fracture patients admitted to a large district hospital between January 1989 to August 2021 were prospectively collected on a hip fracture database using a standardised proforma. Data collected included: age, gender, place of residence prior to admission, American Society of Anaesthesiologist (ASA) score, mini mental test score (MTS) [24], the time of the injury, time of admission and the time of surgery. Mobility was also scored prospectively at the time of admission using a scale from 0 to 9 points as shown in Table 1.

**Table 1:** Mobility assessment tool [25]. Assessed on admission and calculated as the sum of results from the following questions

	Not at all/bedbound	Only with help	On their own with aid	Without difficulty
1. Could they get about the house?	0	1	2	3
2. Was the patient able to get out of the house?	0	1	2	3
3. Could they do their shopping?	0	1	2	3

Patients were included in this study if they were over 60 years old and had sustained a hip fracture requiring operative management. Those patients with pathological fractures, those managed conservatively and patients who were delayed for medical reasons were excluded. Patients were then divided into time categories where time from admission to surgery was under 12 h, 12–24 h, 24–72 h and over 72 h. Separate analysis for time of fall to surgery was performed on the subgroup where time of injury was also available. All patients were followed up with telephone consultation at 12 months and survival was confirmed with either the patient or next of kin. Where patients were not reachable, mortality was confirmed via hospital databases or recorded as missing data.

Count data were summarised as absolute numbers and proportions, continuous data as means and standard deviations. Patient characteristics grouped by time to surgery were compared using Analysis of Variance for the continuous variables and Pearson chi-squared test for the categorical variables. The threshold for statistical significance was set at *p* = 0.05. Multivariate binary logistic regression analysis was performed looking at the odds of 30-day mortality before each delay to surgery threshold versus delay beyond this threshold. All variables in the multivariable logistic regression model were entered without exclusions for collinearity [26]. Data were analysed using R version 3.6.2 (R Foundation for Statistical Computing, Vienna, Austria).

## Results

**Fig. 1:**
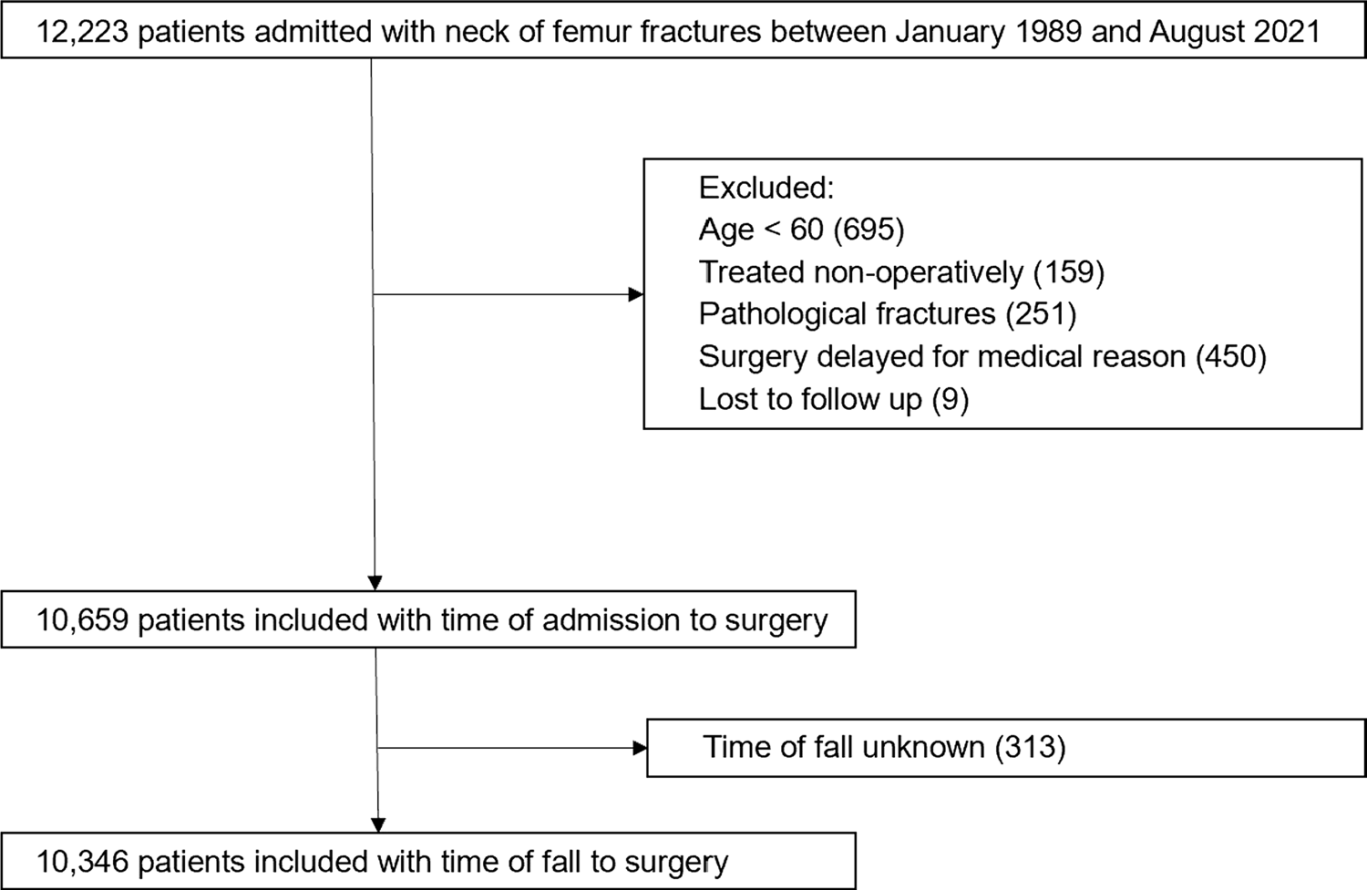
Flow chart showing patient inclusion and exclusion criteria

A total of 12,223 patients were admitted with hip fractures during the study period. Data were available for 10,659 patients after applying exclusion criteria, of which time of fall was available for 10,346 patients (Fig. 1). Mean age for the cohort was 82.2 years (sd 8.39) and 8,023 (75.3%) were female. Mean time from admission to surgery was 27.0 h (sd 28.3) and mean ASA grade 2.71 (sd 0.70). Mean 30-day mortality was 6.2% for the cohort, ranging from 4.8% for those operated in under 12 h from admission to 8.6% for those operated beyond 72 h (Table 2).

**Table 2:** Patient characteristics and mortality—time of admission to surgery

Characteristic	Overall, *N* = 10,659^a^	< 12 h, *N* = 1,226^a^	12–23 h, *N* = 5,192^a^	24–47 h, *N* = 3,311^a^	48–72 h, *N* = 651^a^	> 72 h, *N* = 279^a^	*p*-value
Hours Admission To Surgery	27 (28)	7 (3)	19 (3)	32 (8)	57 (8)	142 (105)	**< 0.001**^b^
Age	82 (8)	82 (9)	82 (8)	82 (8)	82 (8)	83 (9)	0.7^b^
Sex							**0.002**^c^
Female	8,023/10,658 (75%)	932/1,226 (76%)	3,979/5,191 (77%)	2,441/3,311 (74%)	462/651 (71%)	209/279 (75%)	
Male	2,635/10,658 (25%)	294/1,226 (24%)	1,212/5,191 (23%)	870/3,311 (26%)	189/651 (29%)	70/279 (25%)	
Unknown	1	0	1	0	0	0	
Residence							0.4^c^
Institution	2,599/10,659 (24%)	305/1,226 (25%)	1,279/5,192 (25%)	803/3,311 (24%)	157/651 (24%)	55/279 (20%)	
Own home	8,060/10,659 (76%)	921/1,226 (75%)	3,913/5,192 (75%)	2,508/3,311 (76%)	494/651 (76%)	224/279 (80%)	
ASA	2.71 (0.70)	2.61 (0.70)	2.67 (0.70)	2.75 (0.70)	2.89 (0.70)	3.07 (0.75)	**< 0.001**^b^
Unknown	5	0	4	1	0	0	
MTS	6.1 (3.4)	6.1 (3.3)	6.1 (3.4)	6.1 (3.4)	6.0 (3.5)	5.9 (3.4)	> 0.9^b^
Unknown	508	69	252	143	36	8	
Mobility score on admission	6.63 (2.61)	6.64 (2.63)	6.54 (2.62)	6.68 (2.60)	6.88 (2.60)	7.04 (2.55)	**< 0.001**^b^
Unknown	1	0	0	1	0	0	
AO Class							**0.014**^c^
Extracapsular	4,464/10,659 (42%)	542/1,226 (44%)	2,164/5,192 (42%)	1,404/3,311 (42%)	261/651 (40%)	93/279 (33%)	
Intracapsular	6,195/10,659 (58%)	684/1,226 (56%)	3,028/5,192 (58%)	1,907/3,311 (58%)	390/651 (60%)	186/279 (67%)	
Thirty-day mortality							**0.008**^c^
Alive	9,997/10,659 (94%)	1,167/1,226 (95%)	4,891/5,192 (94%)	3,084/3,311 (93%)	600/651 (92%)	255/279 (91%)	
Dead	662/10,659 (6.2%)	59/1,226 (4.8%)	301/5,192 (5.8%)	227/3,311 (6.9%)	51/651 (7.8%)	24/279 (8.6%)	

^a^Mean (SD); *n*/*N* (%)

^b^One-way ANOVA

^c^Pearson's chi-squared test

**Fig. 2:**
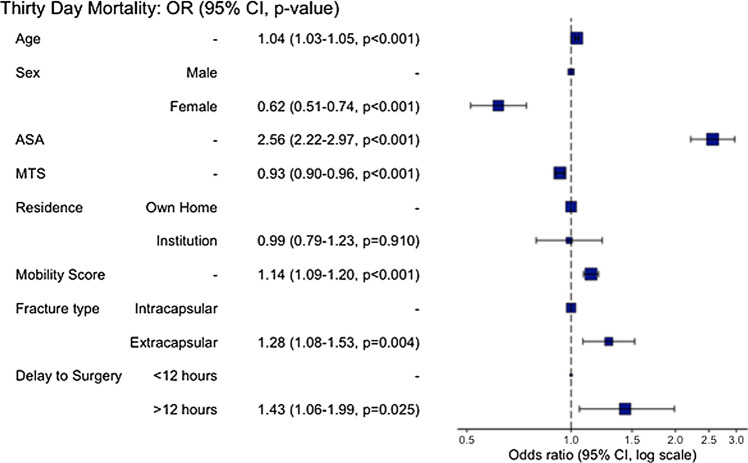
Odds RatioPlot for Delay from Admission to Surgery

Odds of 30-day mortality was 1.43 (CI 1.057–1.988, *p* = 0.025) for delay to surgery from admission of over 12 h compared to under 12 h (Table 3). Odd ratios for 30-day mortality were not significant at any other time threshold. Age, gender, ASA, MTS, mobility score and fracture type remained significant on multivariate analysis (Fig. 2).

**Table 3:** Odds of 30-day mortality for delay to surgery from time of admission

Dependent: 30-day mortality		Alive	Dead	OR (univariable)	OR (multivariable)
Age	Mean	81.9 (8.4)	85.7 (7.8)	1.062 (1.051–1.074, *p* < 0.001)	1.038 (1.026–1.051, *p* < 0.001)
Sex	Male	2408 (91.4%)	227 (8.6%)	–	–
	Female	7588 (94.6%)	435 (5.4%)	0.608 (0.515–0.720, *p* < 0.001)	0.616 (0.513–0.743, *p* < 0.001)
ASA	Mean	2.7 (0.7)	3.2 (0.7)	3.103 (2.735–3.525, *p* < 0.001)	2.562 (2.216–2.967, *p* < 0.001)
MTS	Mean	6.2 (3.4)	4.2 (3.6)	0.858 (0.839–0.877, *p* < 0.001)	0.929 (0.902–0.957, *p* < 0.001)
Residence	Own Home	7676 (95.2%)	384 (4.8%)	–	–
	Institution	2321 (89.3%)	278 (10.7%)	2.394 (2.037–2.812, *p* < 0.001)	0.987 (0.793–1.230, *p* = 0.910)
Mobility Score On Admission	Mean	6.5 (2.6)	8.2 (2.1)	1.325 (1.277–1.375, *p* < 0.001)	1.142 (1.086–1.201, *p* < 0.001)
AO Class	Intracapsular	5861 (94.6%)	334 (5.4%)	–	–
	Extracapsular	4136 (92.7%)	328 (7.3%)	1.392 (1.189–1.629, *p* < 0.001)	1.285 (1.082–1.526, *p* = 0.004)
Delay From Adm12	< 12 h	1167 (95.2%)	59 (4.8%)	–	–
	> 12 h	8830 (93.6%)	603 (6.4%)	1.351 (1.036–1.794, *p* = 0.032)	1.433 (1.057–1.988, *p* = 0.025)

Number in dataframe = 10,659, Number in model = 10,145, Missing = 514, AIC = 4018.1, C-statistic = 0.761, H&L = Chi-sq(8) 9.27 (*p* = 0.320)

**Fig. 3:**
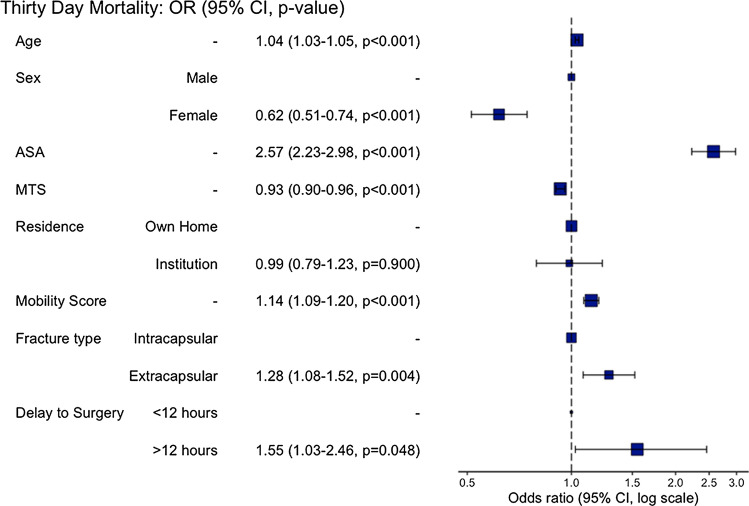
Odds RatioPlot for Delay from Time of Fall to Surgery

Similar demographics were demonstrated for the subgroup where time of fall was available (Table 4). The same variables remained significant on multivariate analysis (Fig. 3). The odds of 30-day mortality for delay to surgery from time of fall were 1.550 (CI 1.026–2.459, *p* = 0.048) at the 12 h threshold (Table 5). This was also not significant at any other time threshold (Table 6).

**Table 4:** Patient characteristics and mortality—time of fall to surgery subgroup

Characteristic	< 12 h, *N* = 693^a^	12–23 h, *N* = 3,461^a^	24–47 h, *N* = 4,405^a^	48–72 h, *N* = 1,033^a^	> 72 h, *N* = 1,067^a^	*p*-value
Time fall to surgery	8 (2)	19 (3)	32 (7)	57 (8)	271 (395)	**< 0.001**^b^
Age	82 (9)	82 (8)	82 (8)	82 (8)	82 (9)	**0.003**^b^
Sex						**< 0.001**^c^
Female	538/693 (78%)	2,674/3,460 (77%)	3,302/4,405 (75%)	748/1,033 (72%)	761/1,067 (71%)	
Male	155/693 (22%)	786/3,460 (23%)	1,103/4,405 (25%)	285/1,033 (28%)	306/1,067 (29%)	
Unknown	0	1	0	0	0	
Residence						**< 0.*001***^*c*^
Institution	171/693 (25%)	737/3,461 (21%)	1,106/4,405 (25%)	276/1,033 (27%)	309/1,067 (29%)	
Own home	522/693 (75%)	2,724/3,461 (79%)	3,299/4,405 (75%)	757/1,033 (73%)	758/1,067 (71%)	
ASA	2.57 (0.72)	2.64 (0.68)	2.72 (0.71)	2.87 (0.69)	2.84 (0.73)	**< 0.001**^b^
Unknown	0	3	2	0	0	
MTS	6.2 (3.3)	6.3 (3.3)	6.0 (3.5)	6.0 (3.5)	5.7 (3.5)	**< 0.001**^b^
Unknown	36	164	212	52	44	
Mobility score on admission	6.47 (2.68)	6.39 (2.57)	6.69 (2.59)	6.92 (2.61)	6.99 (2.72)	**< 0.001**^b^
Unknown	0	0	1	0	0	
AO class						**< 0.001**^c^
Extracapsular	314/693 (45%)	1,514/3,461 (44%)	1,955/4,405 (44%)	404/1,033 (39%)	277/1,067 (26%)	
Intracapsular	379/693 (55%)	1,947/3,461 (56%)	2,450/4,405 (56%)	629/1,033 (61%)	790/1,067 (74%)	
Thirty-day mortality						**0.012**^c^
Alive	665/693 (96%)	3,270/3,461 (94%)	4,110/4,405 (93%)	959/1,033 (93%)	993/1,067 (93%)	
Dead	28/693 (4.0%)	191/3,461 (5.5%)	295/4,405 (6.7%)	74/1,033 (7.2%)	74/1,067 (6.9%)	

^a^Mean (SD); *n*/*N* (%)

^b^One-way ANOVA

^c^Pearson's Chi-squared test

**Table 5:** Odds of 30-day mortality for delay to surgery from time of fall

Dependent: 30-day mortality		Alive	Dead	OR (univariable)	OR (multivariable)
Age	Mean	81.9 (8.4)	85.7 (7.8)	1.062 (1.051–1.074, *p* < 0.001)	1.038 (1.026–1.051, *p* < 0.001)
Sex	Male	2408 (91.4%)	227 (8.6%)	–	–
	Female	7588 (94.6%)	435 (5.4%)	0.608 (0.515–0.720, *p* < 0.001)	0.618 (0.514–0.744, *p* < 0.001)
ASA	Mean	2.7 (0.7)	3.2 (0.7)	3.103 (2.735–3.525, *p* < 0.001)	2.575 (2.227–2.981, *p* < 0.001)
MTS	Mean	6.2 (3.4)	4.2 (3.6)	0.858 (0.839–0.877, *p* < 0.001)	0.929 (0.901–0.956, *p* < 0.001)
Residence	Own Home	7676 (95.2%)	384 (4.8%)	–	–
	Institution	2321 (89.3%)	278 (10.7%)	2.394 (2.037–2.812, *p* < 0.001)	0.986 (0.792–1.228, *p* = 0.900)
Mobility Score On Admission	Mean	6.5 (2.6)	8.2 (2.1)	1.325 (1.277–1.375, *p* < 0.001)	1.141 (1.085–1.200, *p* < 0.001)
AOClass	Intracapsular	5861 (94.6%)	334 (5.4%)	–	–
	Extracapsular	4136 (92.7%)	328 (7.3%)	1.392 (1.189–1.629, *p* < 0.001)	1.284 (1.081–1.525, *p* = 0.004)
Delay From Fall12	< 12 h	665 (96.0%)	28 (4.0%)	–	–
	> 12 h	9332 (93.6%)	634 (6.4%)	1.614 (1.118–2.429, *p* = 0.015)	1.550 (1.026–2.459, *p* = 0.048)

Number in dataframe = 10,659, Number in model = 10,145, Missing = 514, AIC = 4019.1, C-statistic = 0.761, H&L = Chi-sq(8) 9.56 (*p* = 0.297)

**Table 6:** Odds ratios for differing delay to surgery time thresholds

Delay to surgery from admission	OR (univariable)	OR (multivariable)
After 12 h versus within 12 h	1.351 (1.036–1.794, *p* = 0.032)	1.433 (1.057–1.988, *p* = 0.025)
After 24 h versus within 24 h	1.290 (1.101–1.511, *p* = 0.002)	1.128 (0.948–1.341, *p* = 0.172)
After 48 h versus within 48 h	1.366 (1.056–1.743, *p* = 0.015)	1.015 (0.765–1.329, *p* = 0.914)
After 72 h versus within 72 h	1.437 (0.915–2.154, *p* = 0.095)	0.872 (0.526–1.375, *p* = 0.575)

Delay to surgery from fall	OR (univariable)	OR (multivariable)
After 12 h versus within 12 h	1.614 (1.118–2.429, *p* = 0.015)	1.550 (1.026–2.459, *p* = 0.048)
After 24 h versus within 24 h	1.313 (1.113–1.554, *p* = 0.001)	1.086 (0.906–1.306, *p* = 0.375)
After 48 h versus within 48 h	1.187 (0.979–1.430, *p* = 0.076)	0.955 (0.773–1.172, *p* = 0.663
After 72 h versus within 72 h	1.141 (0.882–1.456, *p* = 0.302)	0.931 (0.703–1.216, *p* = 0.610)

## Discussion

National mortality rates for elderly patients with hip fractures have varied considerably in recent years [1], partly as a result of widespread covid-19 infection[27–30] and changes in health service provision during the pandemic. Mortality at our hospital has shown similar yearly variations with a broadly decreasing trend prior to the pandemic. Many factors have influenced this including improvements in peri-operative care and enhanced rehabilitation post-operatively; however, timing of surgery remains a key modifiable factor. This study demonstrates clear benefit from prompt surgical intervention in elderly patients with hip fractures, ideally within 12 h and benefits from a large cohort, minimal loss to follow-up and prospectively collected data. In addition to this, patients delayed for medical reasons were excluded and patient variables known to be correlated with mortality were accounted for with multivariate analysis.

Other studies have suggested differing time thresholds for intervention to reduce mortality; however, conflicting results from these can be explained by small cohorts, heterogenous time categories or incomplete analysis of confounders. More recently, the Hip ATTACK study randomised 2970 patients to early vs standard care; the largest randomised control trial on surgical timing to date and found no difference in mortality rates at 90 days [18].

Best practice tariffs are paid to trusts where surgery is performed within 36 h and NICE guidelines currently recommend surgery on the day of or day after admission. This was based on “pragmatic, organisational and human considerations” after their 2011 evidence review found no specific interval threshold below which reducing delays had no benefit [22]. Outcomes for 30-day mortality in their review were based on evidence from three studies [2, 3, 21].

We also demonstrated similar rates of 30-day mortality when assessed by time of fall rather than admission. Patients with undiagnosed hip fractures may well cope at home for a period of time, suggesting higher functional capacity or minimal initial displacement of fracture, only presenting when their condition deteriorates. Our data suggest patients who are otherwise well should be prioritised by time of admission and the time of fall should not routinely affect decision making. It is also possible that patients present late with long lies due to not being found following a fall and then deteriorate as a result. These would likely have been delayed for medical reasons and therefore excluded from this study or otherwise adjusted for with higher ASA grade.

Covid-19 infection will have also affected mortality rates, particularly during peak infection periods with early variants. Test results for Covid-19 were not included within this study but again these patients will most likely be adjusted for within ASA grade or, if unwell, excluded for delay due to medical reasons.

Whilst our data show the lowest mortality in those operated on within 12 h of admission, we acknowledge that this is dependent on fortuitous arrival time and sufficient theatre capacity. Theatres are not typically staffed to accommodate hip fracture patients overnight for pragmatic reasons, and evidence is conflicting on the impact of out of hours operating on mortality [31–34]. Patients of similar clinical urgency may be prioritised, and it is unlikely the majority of patients could be treated within this timeframe at most hospitals.

Timing of surgery may also have been affected by patients’ clinical condition on presentation or co-morbidities. According to hospital policy at the site where data were collected, surgeries were conducted in the order of presentation to the Emergency Department (ED), unless a patient's medical condition necessitates a delay for optimisation. Patients were excluded from our study where medical delay was documented, reducing large variations in timing due to medical condition. However, we cannot rule out the possibility of insufficient documentation or minor changes, such as the order of patients within an operating list, that could affect the data.

Despite these limitations, our large cohort and detailed accounting for confounding factors show optimising timing of care is crucial to minimising early death as a result of hip fracture.

## Future directions

Our ageing population, whilst driving rising hip fracture incidence, also increases the burden of fragility fractures of the hip. Understanding the impact of delays on these conditions is crucial to guide how best to allocate resources. Our single centre study benefits from consistent data collection and similar peri-operative care across the cohort, but expanding this to a multi-centre study would enhance understanding of this topic. Such expansion would enable a shorter study timeline and diminish the influence of temporal shifts in health policy and practice. Additionally, exploring variations in clinical practice, including differences in clinical skill, departmental resources, and rehabilitation quality across multiple sites, could unveil novel insights. National registries, routine data linkage and artificial intelligence have shown potential to address some of the logistical challenges of collecting large datasets. It is our hope that future studies could benefit from these technological advances in data management to improve the evidence base informing health policy and clinical practice.

## Conclusion

Delay to surgery of over 12 h from admission is associated with a statistically significant increase in 30-day mortality in elderly patients with hip fractures.
